# Management of Chronic Gluteal Follicular Occlusive Disease

**Published:** 2013-07-10

**Authors:** Allison Pagano, Michael J. Feldman

**Affiliations:** Division of Plastic and Reconstructive Surgery, Department of Surgery, Virginia Commonwealth University School of Medicine, Richmond

**Keywords:** hidradenitis suppurativa, dissecting cellulitis, follicular occlusive disease, acne conglobata

**Figure:**
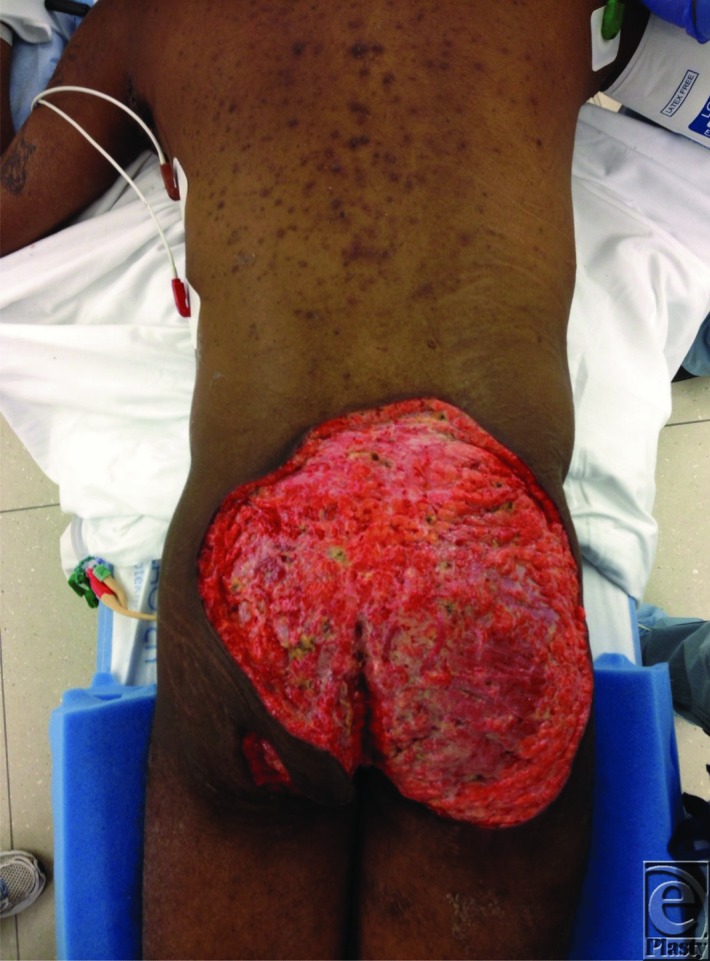


## DESCRIPTION

A 41-year-old African American man has suffered from generalized follicular papules, pustules, and fluctuating nodules since the age of 28. Over the course of 13 years, the patient underwent numerous failed trials of medical treatments in an attempt to control the suppurative, draining sinuses. In August 2012, complete excision and staged reconstruction of the involved areas was recommended.

## QUESTIONS

1. **What is the follicular occlusive triad and in particular, Hidradenitis suppurativa?**
2. **What are Acne Conglobata and Dissecting cellulitis of the scalp?**
3. **What is the pathophysiology of follicular occlusive triad?**
4. **What are the treatment options?**

## DISCUSSION

Follicular occlusion triad is a debilitating chronic cutaneous disorder composed of 3 separate but related diseases. Hidradenitis suppurativa is just one third of the follicular occlusion triad. It is thought to be an androgen-dependent chronic follicular occlusive disease whose pathophysiology is rooted in the apocrine gland. It primarily affects apocrine gland-rich areas such as the axilla, inframammary, perianal, and perigenital regions, and its clinical course is variable. The hallmarks of the disease included recurrent nodules and abscesses with subsequent severe scar formation. Any patient who fits the clinical presentation and is diagnosed with Hidradenitis suppurativa needs to be evaluated for additional evidence of the follicular occlusion triad, which includes Acne Conglobata and Dissecting Cellulitis of the Scalp.

Acne Conglobata, is a form of inflammatory nodulocystic acne most commonly seen in young male individuals, which results in severe scarring primarily affecting the face, buttocks, and trunk. Treatment is similar to that of Hidradenitis, discussed earlier; however, the mainstay of therapy for these patients is low dose isotretinoin. Dissecting cellulitis of the scalp is a disease predominantly in African American male individuals in their second to fourth decade of life. Clinically, these patients develop perifollicular pustules, nodules, and abscesses, with interconnecting sinus tracts that drain pus or blood and often leading to scarring scalp alopecia.'

The triad of diseases is related because of their similar pathophysiology. The initial event shared among the 3 is follicular occlusion caused by abnormal keratinocyte differentiation and shedding. This leads to eventual rupture of the involved follicular gland resulting in release of antigens and inflammatory stimuli. As a result, a combination of periglandular inflammation and cellular proliferation occur and may account for the development of subcutaneous abscesses and sinus tracts that are often chronic and resistant to treatment.

Typical management has included lifestyle modifications such as weight loss, wearing loose fitting clothing, and the avoidance of abrasive washing, however, rarely effective. The first medical treatment option often pursued is topical clindamycin and then a combination of oral antibiotics after failure. In cases of more severe disease, studies have shown significant benefit by combining a short course of oral steroid with long-term oral isotretinoin therapy.^4^ However, as seen in this patient presented, both antibiotic and isotretinoin failure occurs. Biologic therapy including infliximab and adalimumab has shown promise and is particularly useful in patients with coexisting inflammatory bowel disease; however, it must be used with caution. In resistant cases, surgical therapy can be of tremendous value. Surgical excision removing all apocrine glands in the affected area is the recommended form of treatment for severe disease. This ranges from simple incision and drainage to excision and healing by secondary intention, split-thickness grafting, flap closure, and primary closure.

This particular patient's treatment course began with a diverting colostomy because of the extensive perineal and gluteal involvement. The excision and reconstruction were planned in a staged fashion over the course of 4 months. At the time of reconstruction, he was admitted, medically stabilized, and taken to operating room for wide local excision of the perineal and gluteal follicular occlusive disease wounds. Excision extended down to subcutaneous tissue and fascia. At this point, in the hospitalization, the patient's white count peaked to 25.3. Tissue cultures of the wound showed many mixed aerobic and anaerobic bacteria resembling mixed intestinal flora and were negative for fungus. The patient was treated with intravenous vancomycin, levofloxacin, and metronidazole, and the wound was treated open until we confirmed clearance of any residual infection. Once we established control of the wound, it was resurfaced with allograft. The allograft was noted to be full adherent and prepared him for eventual resurfacing with split-thickness autografts a week later. We had to return to the operating room several times to complete the grafting process because of the size of the wound bed. His grafts were protected with tie-over bolster dressings, which facilitated almost complete graft adherence. Graft adherence was also promoted by maintaining a postoperative bed rest protocol in the prone position, which he tolerated well. Following removal of the bolster dressing, the graft sites were treated with Mepitel, lap pads, and ABD pads (CURITY, Mansfield, MA) to fit the wound, held in place with mesh underwear. Pain was controlled with a Dilaudid patient-controlled analgesia, which was weaned to Percocet over the course of several weeks. The patient spent a total of 39 days in the hospital and underwent 5 surgeries to achieve wound closure. Although difficult, it is possible to achieve wound closure in patients with chronic gluteal follicular occlusive disease. Our experience, while limited, has shown success with a staged approach using allograft followed by autograft for wound closure.

## Figures and Tables

**Figure 1:**
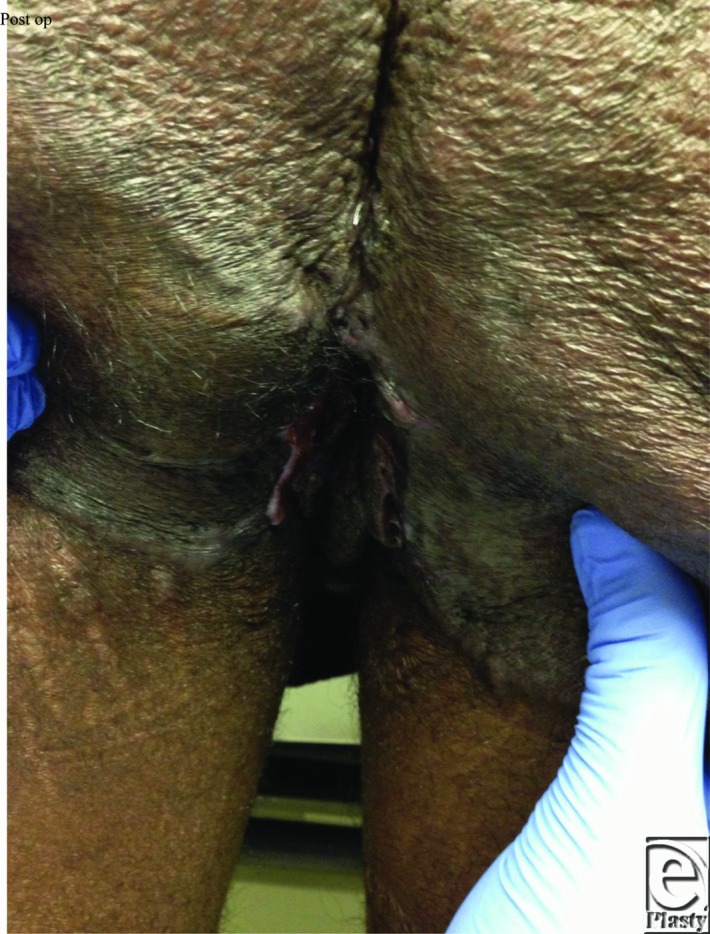
Postoperative wound closure result using the staged approach.

## References

[1] Lee RA, Yoon A, Kist J (2007). Hidradenitis suppurativa: an update. Adv Dermatol.

[2] Self SJ, Montes LF (1970). Follicular occlusion triad. South Med J.

[3] Chicarilli ZN (1987). Follicular occlusion triad: hidradenitis suppurativa, acne conglobata, and dissecting cellulitis of the scalp. Ann Plast Surg.

[4] Wollina U (2013). Acne inversa (Hidradenitis suppurativa): a review with a focus on pathogenesis and treatment. Indian Dermatol Online J.

[5] Rompei R, Petres J (2000). Long-term results of wide surgical excisions in 106 patients with hidradenitis suppurativa. Dermatal Surg.

[6] Wiltz O, Schoetz DJ, Murray JJ (1990). Perianal hidradenitis suppurativa. The Lahey Clinic experience. Dis Colon Rectum.

